# 2-(Biphenyl-4-yl)-5-[3-(4,5,6,7-tetra­hydro­thieno[3,2-*c*]pyridine-5-ylsulfon­yl)thio­phen-2-yl]-1,3,4-oxa­diazole

**DOI:** 10.1107/S1600536811038621

**Published:** 2011-09-30

**Authors:** Hoong-Kun Fun, Madhukar Hemamalini, Sankappa Rai, A. M. Isloor, Prakash Shetty

**Affiliations:** a X-ray Crystallography Unit, School of Physics, Universiti Sains Malaysia, 11800 USM, Penang, Malaysia; b Department of Chemistry, Manipal Institute of Technology, Manipal, India; c Medicinal Chemistry Division, Department of Chemistry, National Institute of Technology, Karnataka, Surathkal, Mangalore 575 025, India; d Department of Printing & Media, Manipal Institute of Technology, Manipal, India

## Abstract

In the title mol­ecule, C_25_H_19_N_3_O_3_S_3_, the tetra­hydro­pyridine ring adopts a half-chair conformation. The dihedral angle between the least-squares plane through the tetra­hydro­pyridine ring and two thio­phene and two benzene rings are 6.25 (9), 89.49 (9), 76.43 (9) and 84.93 (8)°, respectively, while the dihedral angle between the 1,3,4-oxadiazole and tetra­hydro­pyridine rings is 81.14 (9)°. In the crystal, adjacent mol­ecules are connected *via* weak C—H⋯N hydrogen bonds, forming a chain along the *b* axis.

## Related literature

For applications of 4,5,6,7-tetra­hydro­thieno[3,2-*c*]pyridine derivatives, see: Lopez-Rodriguez *et al.* (2001 ▶); Roth *et al.* (1994 ▶); Ying & Rusak (1997 ▶). For a related structure, see: Fun *et al.* (2011 ▶). For ring conformational analysis, see: Cremer & Pople (1975 ▶). For the stability of the temperature controller used in the data collection, see: Cosier & Glazer (1986 ▶). 
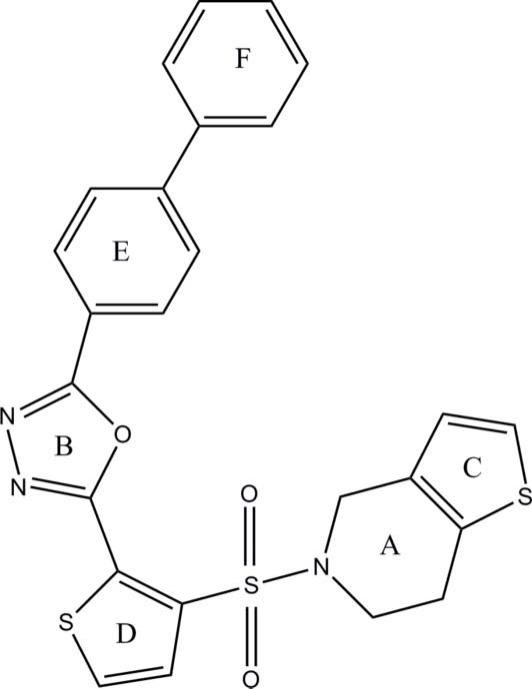


## Experimental

### 

#### Crystal data

C_25_H_19_N_3_O_3_S_3_
*M* *_r_* = 505.61
Triclinic, 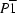

*a* = 7.9108 (1) Å
*b* = 12.0943 (1) Å
*c* = 12.9498 (2) Å
α = 69.253 (1)°
β = 76.794 (1)°
γ = 77.460 (1)°
*V* = 1115.30 (2) Å^3^
*Z* = 2
Mo *K*α radiation
μ = 0.37 mm^−1^
*T* = 100 K
0.33 × 0.16 × 0.09 mm

#### Data collection

Bruker SMART APEXII CCD area-detector diffractometer
Absorption correction: multi-scan (*SADABS*; Bruker, 2009 ▶) *T*_min_ = 0.889, *T*_max_ = 0.966
33050 measured reflections
8887 independent reflections
5904 reflections with *I* > 2σ(*I*)
*R*_int_ = 0.058

#### Refinement

*R*[*F* ^2^ > 2σ(*F* ^2^)] = 0.053
*wR*(*F* ^2^) = 0.122
*S* = 1.03
8887 reflections
307 parameters
H-atom parameters constrained
Δρ_max_ = 0.47 e Å^−3^
Δρ_min_ = −0.56 e Å^−3^

### 

Data collection: *APEX2* (Bruker, 2009 ▶); cell refinement: *SAINT* (Bruker, 2009 ▶); data reduction: *SAINT*; program(s) used to solve structure: *SHELXTL* (Sheldrick, 2008 ▶); program(s) used to refine structure: *SHELXTL*; molecular graphics: *SHELXTL*; software used to prepare material for publication: *SHELXTL* and *PLATON* (Spek, 2009 ▶).

## Supplementary Material

Crystal structure: contains datablock(s) global, I. DOI: 10.1107/S1600536811038621/tk2792sup1.cif

Structure factors: contains datablock(s) I. DOI: 10.1107/S1600536811038621/tk2792Isup2.hkl

Supplementary material file. DOI: 10.1107/S1600536811038621/tk2792Isup3.cml

Additional supplementary materials:  crystallographic information; 3D view; checkCIF report

## Figures and Tables

**Table 1:** Hydrogen-bond geometry (Å, °)

*D*—H⋯*A*	*D*—H	H⋯*A*	*D*⋯*A*	*D*—H⋯*A*
C24—H24*A*⋯N1^i^	0.99	2.52	3.417 (2)	150

Symmetry code: (i) 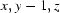.

**Fig. 1.**
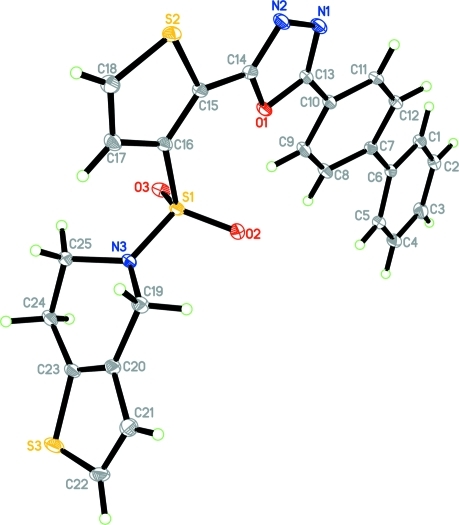
The molecular structure of the title compound, showing 30% probability displacement ellipsoids.

**Fig. 2.**
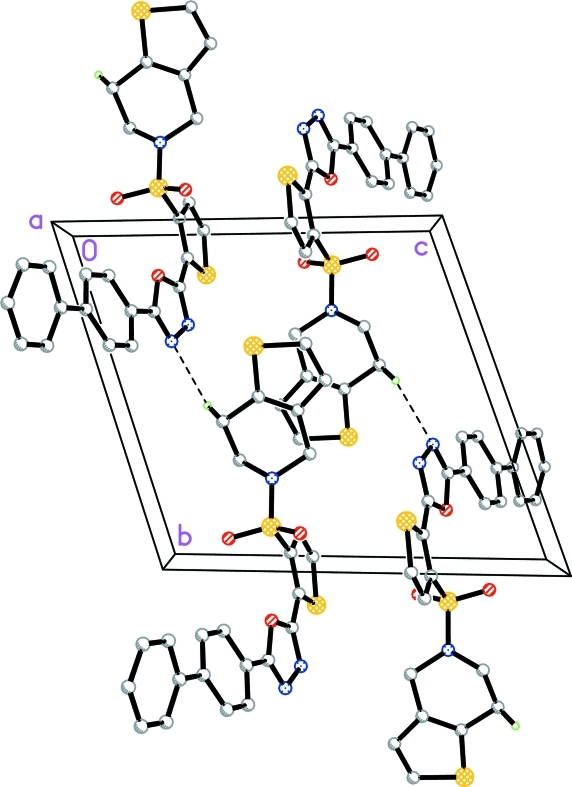
A view of the crystal packing of the title compound (I). H atoms not involved in hydrogen bonding are omitted.

## supplementary crystallographic information

### Comment

4,5,6,7-Tetrahydrothieno[3,2-c]pyridine derivatives have been extensively studied in medicinal chemistry due to their various biological activities (Lopez-Rodriguez *et al.*, 2001). 4,5,6,7-Tetrahydrothieno[3,2-c] pyridine oxadiazole derivatives are mainly used in CNS functions and disorders such as schizophrenia (Roth *et al.*, 1994), depression, epilepsy, migraine, and control of circadian rhythm (Ying & Rusak, 1997). Keeping in view of the biological importance of this class of compound, we synthesized the title compound to study its X-ray crystal structure.

In the title compound (Fig. 1), the rings A (N3/C19,C20,C23–C25), B (N1/N2/O1/C13,C14), C (S3/C20–C23), D (S2/C15–C18) E (C7–C12) and F (C1–C6) are essentially planar. The tetrahydropyridine (N3/C19,C20, C23–C25) ring adopts a half-chair conformation with puckering parameters Q = 0.4970 (18) Å, θ = 129.3 (2)° and φ = 153.0 (3)° . The dihedral angle between the least-square planes of the rings are A/B = 81.14 (9)°, A/C = 6.25 (9)°, A/D = 89.49 (9)°, A/E = 84.93 (8)°, A/F = 76.43 (9)° B/C = 78.71 (10)°, B/D = 9.55 (10)°, B/E = 10.88 (9)°, B/F = 11.16 (10)°, C/D = 87.86 (9)°, C/E = 83.55 (9)°, C/F = 73.04 (9)°, D/E = 13.31 (9)° and D/F = 16.40 (9)°.

In the crystal structure, (Fig. 2), adjacent molecules are connected *via* weak intermolecular C—H···N (Table 1) hydrogen bonds to form one-dimensional chains along the *b*-axis.

### Experimental

To a mixture of 3-(6,7-dihydrothieno[3,2-c]pyridine-5(4*H*)-ylsulfonyl) thiophene-2-carbohydrazide (0.5 g, 0.0014 mol) and biphenyl carboxylic acid (0.28 g, 0.0014 mol), neutral alumina (0.5 g) and POCl_3_ (1.1 g, 0.007 mol) were added. The resulting mixture was irradiated in a microwave oven for 5 min. Mass analysis of crude reaction mixture confirmed the completion of the reaction. The reaction mixture was concentrated and the residue was purified by column chromatography to get the title compound, which was recrystallised using acetone. Yield: 68%, m.p. 441–443 K.

### Refinement

All hydrogen atoms were positioned geometrically [C–H = 0.95–0.99 Å] and were refined using a riding model, with *U*_iso_(H) = 1.2 *U*_eq_(C).

### Crystal data

**Table:**

C_25_H_19_N_3_O_3_S_3_	*Z* = 2
*M**_r_* = 505.61	*F*(000) = 524
Triclinic, *P*1	*D*_x_ = 1.506 Mg m^−^^3^
Hall symbol: -P 1	Mo *K*α radiation, λ = 0.71073 Å
*a* = 7.9108 (1) Å	Cell parameters from 6236 reflections
*b* = 12.0943 (1) Å	θ = 2.7–33.5°
*c* = 12.9498 (2) Å	µ = 0.37 mm^−^^1^
α = 69.253 (1)°	*T* = 100 K
β = 76.794 (1)°	Block, colourless
γ = 77.460 (1)°	0.33 × 0.16 × 0.09 mm
*V* = 1115.30 (2) Å^3^	

### Data collection

**Table:**

Bruker SMART APEXII CCD area-detector diffractometer	8887 independent reflections
Radiation source: fine-focus sealed tube	5904 reflections with *I* > 2σ(*I*)
graphite	*R*_int_ = 0.058
φ and ω scans	θ_max_ = 33.8°, θ_min_ = 1.8°
Absorption correction: multi-scan (*SADABS*; Bruker, 2009)	*h* = −12→12
*T*_min_ = 0.889, *T*_max_ = 0.966	*k* = −18→18
33050 measured reflections	*l* = −19→20

### Refinement

**Table:**

Refinement on *F*^2^	Primary atom site location: structure-invariant direct methods
Least-squares matrix: full	Secondary atom site location: difference Fourier map
*R*[*F*^2^ > 2σ(*F*^2^)] = 0.053	Hydrogen site location: inferred from neighbouring sites
*wR*(*F*^2^) = 0.122	H-atom parameters constrained
*S* = 1.03	*w* = 1/[σ^2^(*F*_o_^2^) + (0.0478*P*)^2^ + 0.3497*P*] where *P* = (*F*_o_^2^ + 2*F*_c_^2^)/3
8887 reflections	(Δ/σ)_max_ = 0.001
307 parameters	Δρ_max_ = 0.47 e Å^−^^3^
0 restraints	Δρ_min_ = −0.56 e Å^−^^3^

### Special details

**Table:**

Experimental. The crystal was placed in the cold stream of an Oxford Cryosystems Cobra open-flow nitrogen cryostat (Cosier & Glazer, 1986) operating at 100.0 (1) K.
Geometry. All s.u.'s (except the s.u. in the dihedral angle between two l.s. planes) are estimated using the full covariance matrix. The cell s.u.'s are taken into account individually in the estimation of s.u.'s in distances, angles and torsion angles; correlations between s.u.'s in cell parameters are only used when they are defined by crystal symmetry. An approximate (isotropic) treatment of cell s.u.'s is used for estimating s.u.'s involving l.s. planes.
Refinement. Refinement of F^2^ against ALL reflections. The weighted R-factor wR and goodness of fit S are based on F^2^, conventional R-factors R are based on F, with F set to zero for negative F^2^. The threshold expression of F^2^ > 2σ(F^2^) is used only for calculating R-factors(gt) etc. and is not relevant to the choice of reflections for refinement. R-factors based on F^2^ are statistically about twice as large as those based on F, and R- factors based on ALL data will be even larger.

### Fractional atomic coordinates and isotropic or equivalent isotropic displacement parameters (Å^2^)

**Table:**

	*x*	*y*	*z*	*U*_iso_*/*U*_eq_	
S1	0.68865 (5)	0.88728 (4)	0.30396 (4)	0.01750 (9)	
S2	0.21890 (5)	1.13555 (4)	0.34716 (4)	0.02341 (10)	
S3	0.87479 (6)	0.36589 (4)	0.41597 (5)	0.03181 (13)	
O1	0.72380 (15)	1.15330 (10)	0.22602 (10)	0.0197 (2)	
O2	0.79409 (15)	0.90109 (11)	0.37398 (11)	0.0232 (3)	
O3	0.74563 (16)	0.92047 (11)	0.18551 (11)	0.0230 (3)	
N1	0.66367 (19)	1.34718 (13)	0.20268 (14)	0.0253 (3)	
N2	0.51049 (19)	1.29548 (13)	0.25770 (14)	0.0250 (3)	
N3	0.66204 (18)	0.74881 (12)	0.34721 (12)	0.0185 (3)	
C1	1.5791 (2)	1.37068 (16)	−0.08077 (16)	0.0227 (3)	
H1A	1.5056	1.4454	−0.0869	0.027*	
C2	1.7532 (2)	1.36806 (17)	−0.13241 (16)	0.0252 (4)	
H2A	1.7985	1.4409	−0.1724	0.030*	
C3	1.8621 (2)	1.25980 (18)	−0.12625 (18)	0.0293 (4)	
H3A	1.9800	1.2582	−0.1643	0.035*	
C4	1.7966 (2)	1.15442 (17)	−0.06408 (18)	0.0290 (4)	
H4A	1.8711	1.0801	−0.0579	0.035*	
C5	1.6239 (2)	1.15597 (16)	−0.01085 (16)	0.0232 (4)	
H5A	1.5816	1.0827	0.0323	0.028*	
C6	1.5102 (2)	1.26432 (15)	−0.01967 (15)	0.0201 (3)	
C7	1.3224 (2)	1.26503 (15)	0.03247 (14)	0.0192 (3)	
C8	1.2404 (2)	1.16451 (16)	0.05717 (16)	0.0237 (4)	
H8A	1.3061	1.0961	0.0392	0.028*	
C9	1.0665 (2)	1.16279 (16)	0.10701 (16)	0.0233 (4)	
H9A	1.0143	1.0934	0.1239	0.028*	
C10	0.9670 (2)	1.26333 (15)	0.13265 (15)	0.0197 (3)	
C11	1.0453 (2)	1.36463 (15)	0.10693 (16)	0.0214 (3)	
H11A	0.9782	1.4338	0.1228	0.026*	
C12	1.2206 (2)	1.36495 (15)	0.05825 (15)	0.0208 (3)	
H12A	1.2727	1.4343	0.0421	0.025*	
C13	0.7844 (2)	1.26086 (15)	0.18576 (15)	0.0195 (3)	
C14	0.5534 (2)	1.18253 (15)	0.26865 (15)	0.0197 (3)	
C15	0.4398 (2)	1.09154 (15)	0.31380 (15)	0.0186 (3)	
C16	0.4768 (2)	0.97000 (15)	0.32871 (15)	0.0188 (3)	
C17	0.3231 (2)	0.91496 (16)	0.36701 (16)	0.0242 (4)	
H17A	0.3234	0.8322	0.3819	0.029*	
C18	0.1748 (2)	0.99439 (16)	0.38000 (17)	0.0256 (4)	
H18A	0.0600	0.9732	0.4046	0.031*	
C19	0.6485 (2)	0.67707 (16)	0.46603 (15)	0.0224 (3)	
H19A	0.5237	0.6786	0.5020	0.027*	
H19B	0.7095	0.7098	0.5051	0.027*	
C20	0.7328 (2)	0.55051 (15)	0.47309 (16)	0.0218 (3)	
C21	0.7922 (2)	0.46336 (18)	0.56962 (18)	0.0300 (4)	
H21A	0.7774	0.4762	0.6396	0.036*	
C22	0.8730 (2)	0.35916 (18)	0.5505 (2)	0.0353 (5)	
H22A	0.9222	0.2912	0.6051	0.042*	
C23	0.7665 (2)	0.51108 (15)	0.38342 (16)	0.0220 (3)	
C24	0.7232 (2)	0.58363 (15)	0.26954 (16)	0.0232 (4)	
H24A	0.6660	0.5368	0.2420	0.028*	
H24B	0.8321	0.6043	0.2163	0.028*	
C25	0.5997 (2)	0.69765 (15)	0.27725 (16)	0.0215 (3)	
H25A	0.5965	0.7564	0.2013	0.026*	
H25B	0.4793	0.6791	0.3106	0.026*	

### Atomic displacement parameters (Å^2^)

**Table:**

	*U*^11^	*U*^22^	*U*^33^	*U*^12^	*U*^13^	*U*^23^
S1	0.01654 (16)	0.01276 (18)	0.0226 (2)	−0.00104 (13)	−0.00308 (15)	−0.00578 (16)
S2	0.01898 (18)	0.0208 (2)	0.0301 (3)	0.00215 (15)	−0.00360 (17)	−0.01094 (19)
S3	0.0246 (2)	0.0142 (2)	0.0525 (3)	0.00070 (16)	−0.0087 (2)	−0.0063 (2)
O1	0.0199 (5)	0.0135 (6)	0.0258 (7)	−0.0021 (4)	−0.0021 (5)	−0.0078 (5)
O2	0.0226 (6)	0.0184 (6)	0.0317 (7)	−0.0033 (5)	−0.0089 (5)	−0.0088 (5)
O3	0.0250 (6)	0.0167 (6)	0.0232 (7)	−0.0003 (5)	−0.0006 (5)	−0.0051 (5)
N1	0.0236 (7)	0.0159 (7)	0.0382 (10)	−0.0021 (5)	−0.0056 (6)	−0.0108 (7)
N2	0.0219 (7)	0.0157 (7)	0.0392 (10)	−0.0024 (5)	−0.0043 (6)	−0.0118 (7)
N3	0.0232 (6)	0.0120 (6)	0.0207 (7)	−0.0023 (5)	−0.0059 (5)	−0.0046 (6)
C1	0.0264 (8)	0.0192 (8)	0.0242 (9)	−0.0018 (6)	−0.0054 (7)	−0.0091 (7)
C2	0.0290 (8)	0.0233 (9)	0.0263 (10)	−0.0076 (7)	−0.0032 (7)	−0.0102 (8)
C3	0.0225 (8)	0.0309 (10)	0.0386 (12)	−0.0037 (7)	−0.0016 (8)	−0.0184 (9)
C4	0.0249 (8)	0.0232 (9)	0.0426 (12)	0.0038 (7)	−0.0097 (8)	−0.0167 (9)
C5	0.0258 (8)	0.0179 (8)	0.0278 (10)	−0.0006 (6)	−0.0090 (7)	−0.0083 (7)
C6	0.0246 (7)	0.0177 (8)	0.0208 (9)	−0.0016 (6)	−0.0083 (6)	−0.0076 (7)
C7	0.0242 (7)	0.0156 (8)	0.0177 (8)	−0.0027 (6)	−0.0055 (6)	−0.0042 (7)
C8	0.0277 (8)	0.0169 (8)	0.0281 (10)	−0.0029 (6)	−0.0025 (7)	−0.0107 (7)
C9	0.0275 (8)	0.0156 (8)	0.0288 (10)	−0.0050 (6)	−0.0038 (7)	−0.0090 (7)
C10	0.0232 (7)	0.0155 (8)	0.0213 (9)	−0.0027 (6)	−0.0053 (6)	−0.0060 (7)
C11	0.0255 (8)	0.0126 (7)	0.0260 (9)	−0.0008 (6)	−0.0057 (7)	−0.0062 (7)
C12	0.0243 (8)	0.0142 (8)	0.0241 (9)	−0.0041 (6)	−0.0046 (7)	−0.0053 (7)
C13	0.0229 (7)	0.0134 (7)	0.0237 (9)	−0.0027 (6)	−0.0070 (6)	−0.0060 (7)
C14	0.0195 (7)	0.0177 (8)	0.0228 (9)	0.0009 (6)	−0.0058 (6)	−0.0084 (7)
C15	0.0192 (7)	0.0165 (8)	0.0204 (8)	−0.0003 (6)	−0.0035 (6)	−0.0075 (7)
C16	0.0182 (7)	0.0158 (8)	0.0218 (9)	−0.0007 (6)	−0.0032 (6)	−0.0065 (7)
C17	0.0208 (7)	0.0187 (8)	0.0323 (10)	−0.0048 (6)	−0.0009 (7)	−0.0082 (8)
C18	0.0203 (7)	0.0232 (9)	0.0311 (10)	−0.0043 (7)	0.0003 (7)	−0.0080 (8)
C19	0.0258 (8)	0.0191 (8)	0.0216 (9)	−0.0048 (6)	−0.0029 (7)	−0.0052 (7)
C20	0.0199 (7)	0.0161 (8)	0.0262 (9)	−0.0056 (6)	−0.0056 (7)	−0.0001 (7)
C21	0.0296 (9)	0.0261 (10)	0.0310 (11)	−0.0134 (7)	−0.0100 (8)	0.0032 (8)
C22	0.0275 (9)	0.0190 (9)	0.0505 (14)	−0.0079 (7)	−0.0179 (9)	0.0094 (9)
C23	0.0174 (7)	0.0134 (8)	0.0328 (10)	−0.0033 (6)	−0.0038 (7)	−0.0042 (7)
C24	0.0266 (8)	0.0163 (8)	0.0281 (10)	−0.0031 (6)	−0.0039 (7)	−0.0091 (7)
C25	0.0252 (8)	0.0169 (8)	0.0248 (9)	−0.0021 (6)	−0.0095 (7)	−0.0070 (7)

### Geometric parameters (Å, °)

**Table:**

S1—O3	1.4295 (13)	C8—C9	1.381 (2)
S1—O2	1.4350 (12)	C8—H8A	0.9500
S1—N3	1.6116 (14)	C9—C10	1.400 (2)
S1—C16	1.7793 (16)	C9—H9A	0.9500
S2—C18	1.7012 (19)	C10—C11	1.393 (2)
S2—C15	1.7138 (16)	C10—C13	1.453 (2)
S3—C22	1.712 (3)	C11—C12	1.387 (2)
S3—C23	1.7270 (17)	C11—H11A	0.9500
O1—C14	1.3561 (19)	C12—H12A	0.9500
O1—C13	1.367 (2)	C14—C15	1.447 (2)
N1—C13	1.298 (2)	C15—C16	1.386 (2)
N1—N2	1.409 (2)	C16—C17	1.417 (2)
N2—C14	1.298 (2)	C17—C18	1.365 (2)
N3—C19	1.467 (2)	C17—H17A	0.9500
N3—C25	1.477 (2)	C18—H18A	0.9500
C1—C2	1.386 (2)	C19—C20	1.510 (2)
C1—C6	1.401 (2)	C19—H19A	0.9900
C1—H1A	0.9500	C19—H19B	0.9900
C2—C3	1.389 (3)	C20—C23	1.357 (3)
C2—H2A	0.9500	C20—C21	1.419 (3)
C3—C4	1.382 (3)	C21—C22	1.363 (3)
C3—H3A	0.9500	C21—H21A	0.9500
C4—C5	1.382 (3)	C22—H22A	0.9500
C4—H4A	0.9500	C23—C24	1.499 (3)
C5—C6	1.403 (2)	C24—C25	1.528 (2)
C5—H5A	0.9500	C24—H24A	0.9900
C6—C7	1.484 (2)	C24—H24B	0.9900
C7—C12	1.400 (2)	C25—H25A	0.9900
C7—C8	1.405 (2)	C25—H25B	0.9900
			
O3—S1—O2	119.80 (8)	C7—C12—H12A	119.4
O3—S1—N3	107.36 (7)	N1—C13—O1	112.21 (15)
O2—S1—N3	107.62 (7)	N1—C13—C10	129.83 (16)
O3—S1—C16	107.66 (8)	O1—C13—C10	117.95 (14)
O2—S1—C16	107.45 (7)	N2—C14—O1	112.94 (15)
N3—S1—C16	106.22 (8)	N2—C14—C15	127.56 (15)
C18—S2—C15	92.10 (8)	O1—C14—C15	119.40 (14)
C22—S3—C23	91.92 (9)	C16—C15—C14	130.54 (15)
C14—O1—C13	102.72 (12)	C16—C15—S2	111.12 (12)
C13—N1—N2	106.34 (14)	C14—C15—S2	118.16 (12)
C14—N2—N1	105.78 (14)	C15—C16—C17	112.11 (14)
C19—N3—C25	114.76 (13)	C15—C16—S1	126.07 (13)
C19—N3—S1	121.74 (11)	C17—C16—S1	121.82 (13)
C25—N3—S1	121.27 (12)	C18—C17—C16	112.37 (16)
C2—C1—C6	120.71 (16)	C18—C17—H17A	123.8
C2—C1—H1A	119.6	C16—C17—H17A	123.8
C6—C1—H1A	119.6	C17—C18—S2	112.29 (13)
C1—C2—C3	120.59 (18)	C17—C18—H18A	123.9
C1—C2—H2A	119.7	S2—C18—H18A	123.9
C3—C2—H2A	119.7	N3—C19—C20	107.80 (14)
C4—C3—C2	119.14 (17)	N3—C19—H19A	110.1
C4—C3—H3A	120.4	C20—C19—H19A	110.1
C2—C3—H3A	120.4	N3—C19—H19B	110.1
C5—C4—C3	120.74 (17)	C20—C19—H19B	110.1
C5—C4—H4A	119.6	H19A—C19—H19B	108.5
C3—C4—H4A	119.6	C23—C20—C21	113.21 (17)
C4—C5—C6	120.89 (17)	C23—C20—C19	122.01 (16)
C4—C5—H5A	119.6	C21—C20—C19	124.71 (17)
C6—C5—H5A	119.6	C22—C21—C20	112.31 (19)
C1—C6—C5	117.87 (16)	C22—C21—H21A	123.8
C1—C6—C7	121.56 (15)	C20—C21—H21A	123.8
C5—C6—C7	120.56 (16)	C21—C22—S3	111.67 (16)
C12—C7—C8	117.65 (15)	C21—C22—H22A	124.2
C12—C7—C6	121.74 (15)	S3—C22—H22A	124.2
C8—C7—C6	120.61 (15)	C20—C23—C24	125.40 (15)
C9—C8—C7	121.50 (15)	C20—C23—S3	110.88 (14)
C9—C8—H8A	119.2	C24—C23—S3	123.68 (13)
C7—C8—H8A	119.2	C23—C24—C25	108.88 (14)
C8—C9—C10	120.02 (16)	C23—C24—H24A	109.9
C8—C9—H9A	120.0	C25—C24—H24A	109.9
C10—C9—H9A	120.0	C23—C24—H24B	109.9
C11—C10—C9	119.27 (15)	C25—C24—H24B	109.9
C11—C10—C13	120.89 (15)	H24A—C24—H24B	108.3
C9—C10—C13	119.84 (15)	N3—C25—C24	109.48 (13)
C12—C11—C10	120.28 (15)	N3—C25—H25A	109.8
C12—C11—H11A	119.9	C24—C25—H25A	109.8
C10—C11—H11A	119.9	N3—C25—H25B	109.8
C11—C12—C7	121.25 (16)	C24—C25—H25B	109.8
C11—C12—H12A	119.4	H25A—C25—H25B	108.2
			
C13—N1—N2—C14	−0.1 (2)	C13—O1—C14—C15	176.18 (15)
O3—S1—N3—C19	163.96 (12)	N2—C14—C15—C16	−179.39 (18)
O2—S1—N3—C19	33.75 (15)	O1—C14—C15—C16	4.5 (3)
C16—S1—N3—C19	−81.09 (14)	N2—C14—C15—S2	5.9 (3)
O3—S1—N3—C25	−33.90 (14)	O1—C14—C15—S2	−170.19 (12)
O2—S1—N3—C25	−164.11 (12)	C18—S2—C15—C16	−0.32 (14)
C16—S1—N3—C25	81.06 (14)	C18—S2—C15—C14	175.35 (15)
C6—C1—C2—C3	1.2 (3)	C14—C15—C16—C17	−174.82 (18)
C1—C2—C3—C4	−2.6 (3)	S2—C15—C16—C17	0.16 (19)
C2—C3—C4—C5	1.6 (3)	C14—C15—C16—S1	5.0 (3)
C3—C4—C5—C6	0.8 (3)	S2—C15—C16—S1	179.99 (10)
C2—C1—C6—C5	1.2 (3)	O3—S1—C16—C15	−71.56 (17)
C2—C1—C6—C7	−177.54 (16)	O2—S1—C16—C15	58.74 (17)
C4—C5—C6—C1	−2.2 (3)	N3—S1—C16—C15	173.69 (15)
C4—C5—C6—C7	176.57 (16)	O3—S1—C16—C17	108.25 (16)
C1—C6—C7—C12	−22.9 (3)	O2—S1—C16—C17	−121.45 (15)
C5—C6—C7—C12	158.41 (17)	N3—S1—C16—C17	−6.50 (17)
C1—C6—C7—C8	156.82 (17)	C15—C16—C17—C18	0.1 (2)
C5—C6—C7—C8	−21.9 (3)	S1—C16—C17—C18	−179.69 (14)
C12—C7—C8—C9	−1.2 (3)	C16—C17—C18—S2	−0.4 (2)
C6—C7—C8—C9	179.15 (17)	C15—S2—C18—C17	0.41 (16)
C7—C8—C9—C10	0.9 (3)	C25—N3—C19—C20	50.82 (18)
C8—C9—C10—C11	0.3 (3)	S1—N3—C19—C20	−145.95 (12)
C8—C9—C10—C13	−179.58 (17)	N3—C19—C20—C23	−16.1 (2)
C9—C10—C11—C12	−1.1 (3)	N3—C19—C20—C21	160.65 (16)
C13—C10—C11—C12	178.72 (17)	C23—C20—C21—C22	0.9 (2)
C10—C11—C12—C7	0.8 (3)	C19—C20—C21—C22	−176.09 (16)
C8—C7—C12—C11	0.3 (3)	C20—C21—C22—S3	−0.7 (2)
C6—C7—C12—C11	179.98 (16)	C23—S3—C22—C21	0.29 (15)
N2—N1—C13—O1	−0.2 (2)	C21—C20—C23—C24	−178.48 (16)
N2—N1—C13—C10	−179.90 (17)	C19—C20—C23—C24	−1.4 (3)
C14—O1—C13—N1	0.41 (19)	C21—C20—C23—S3	−0.71 (19)
C14—O1—C13—C10	−179.86 (15)	C19—C20—C23—S3	176.41 (13)
C11—C10—C13—N1	10.6 (3)	C22—S3—C23—C20	0.25 (14)
C9—C10—C13—N1	−169.55 (18)	C22—S3—C23—C24	178.07 (15)
C11—C10—C13—O1	−169.08 (15)	C20—C23—C24—C25	−12.7 (2)
C9—C10—C13—O1	10.8 (2)	S3—C23—C24—C25	169.83 (12)
N1—N2—C14—O1	0.4 (2)	C19—N3—C25—C24	−67.81 (18)
N1—N2—C14—C15	−175.96 (17)	S1—N3—C25—C24	128.87 (14)
C13—O1—C14—N2	−0.48 (19)	C23—C24—C25—N3	43.51 (19)

### Hydrogen-bond geometry (Å, °)

**Table:**

*D*—H···*A*	*D*—H	H···*A*	*D*···*A*	*D*—H···*A*
C24—H24A···N1^i^	0.99	2.52	3.417 (2)	150

Symmetry codes: (i) *x*, *y*−1, *z*.

## References

[bb1] Bruker (2009). *APEX2*, *SAINT* and *SADABS* Bruker AXS Inc., Madison, Wisconsin, USA.

[bb2] Cosier, J. & Glazer, A. M. (1986). *J. Appl. Cryst.* **19**, 105–107.

[bb3] Cremer, D. & Pople, J. A. (1975). *J. Am. Chem. Soc* **97**, 1354–1358.

[bb9] Fun, H.-K., Hemamalini, M., Rai, S., Isloor, A. M. & Shetty, P. (2011). *Acta Cryst.* E**67**, o2743–o2744. 10.1107/S1600536811038529 PMC3201536 22058804

[bb4] Lopez-Rodriguez, M. L., Murcia, M., Benhamu, B., Viso, A., Campillo, M. & Pardo, L. (2001). *Bioorg. Med. Chem. Lett.* **11**, 2807–2811. 10.1016/s0960-894x(01)00517-0 11597405

[bb5] Roth, B. L., Craigo, S. C., Choudhary, M. S., Uluer, A., Monsma, F. J. Jr, Shen, Y., Meltzer, H. Y. & Sibley, D. R. (1994). *J. Pharm. Exp. Ther.* **268**, 1403–1410. 7908055

[bb6] Sheldrick, G. M. (2008). *Acta Cryst.* A**64**, 112–122. 10.1107/S0108767307043930 18156677

[bb7] Spek, A. L. (2009). *Acta Cryst.* D**65**, 148–155. 10.1107/S090744490804362X PMC2631630 19171970

[bb8] Ying, S. W. & Rusak, B. (1997). *Brain Res.* **755**, 246–254. 10.1016/s0006-8993(97)00102-9 9175892

